# Genassemblage 2.0 software facilitates conservation of genetic variation of captively propagated species

**DOI:** 10.1038/s41598-020-74683-9

**Published:** 2020-10-21

**Authors:** Dariusz Kaczmarczyk, Jacek Wolnicki

**Affiliations:** 1 Department of Environmental Biotechnology, University of Warmia and Mazury in Olsztyn, Słoneczna 45G, 10-718 Olsztyn, Poland; 2 Pond Fishery Department, Inland Fisheries Institute in Olsztyn, Oczapowskiego 10, 10-719 Olsztyn, Poland

**Keywords:** Biodiversity, Conservation biology, Molecular ecology, Computational science, Software

## Abstract

In conservation of captively propagated species, conserving genetic diversity is important. Here, we present an example of the use of Genassemblage 2.0 software in conserving the genetic variation of the lake minnow (*Eupallasella percnurus*). This fish has low genetic variation and is at risk of extinction in the western edge of its range, which includes Poland. Fish from one Polish population were captured (23 males, 25 females). Fin clips were taken, and DNA was extracted. Polymorphic microsatellites (13) were used to prepare genetic profiles, assess genetic variation in the fish and estimate genetic diversity in their progeny. Alleles were scored using an automatic capillary sequencer. The four and eight best variants of spawning pairs, and the optimal sets for group volitional breeding (four males, four females; eight males, eight females) were identified using Genassemblage 2.0. In the sets of 8 and 16 fish for group breeding, the mean heterozygosity, the number of alleles, and the share of “weak” heterozygotes (0.493, 24, 0.239 and 0.479, 23, 0.257, respectively) were better than the mean values for the progeny of all potential breeding pairs. For group volitional breeding, one set of four males and four females, and numerous sets of eight males and eight females would enable transmission of all 33 alleles identified in the potential broodstock and an expected progeny heterozygosity of 0.441 and 0.414, respectively. These expected heterozygosity values are higher than those in the broodstock. For practical purposes, the larger sets would be preferable for avoiding a future inbreeding and genetic drift.

## Introduction

Maintaining high genetic variation, as measured by high heterozygosity and allelic diversity, is crucial for keeping a population viable and able to adapt to environmental changes^1–3^. Conserving genetic diversity is important in conservation of captively propagated species. Unfortunately, genetic variation within captively propagated populations may decrease^4–6^ due to (1) obtaining large groups of juveniles from a few parental individuals, (2) pairing individuals without knowledge of the genetic differences between them, (3) possible inbreeding events^7^, or (4) domestication of the broodstock^8^. A decrease in population genetic variation may reduce that population’s viability and its potential to adapt to environmental stresses^3^. Guidelines have been developed to inform broodstock collection, mating, rearing of young, and outplanting^9^.

An example of a species that is at risk of experiencing reduced genetic variation is the lake minnow, *Eupallasella percnurus* (Pallas, 1814), a cyprinid fish. Its populations occur in the northern hemisphere from Poland to the Pacific coast^10^. In the western part of its range, this species is in critical danger of extinction due to the specific nature of its habitats, which are small and shallow water bodies that exist for only several decades^11^. In Poland, the lake minnow is under strict protection, involving active conservation measures^12^. One of the most common measures used so far is outplanting of juvenile fish produced in aquaculture facilities to selected water bodies to establish new populations^13^.

One way of addressing the problem of potential loss of genetic variation in such populations is by preparing genetic profiles of individuals by screening highly polymorphic fragments of DNA such as microsatellites. With these profiles, individuals that are as different as possible can be identified and assembled in to breeding pairs^14^. To use the information in these genetic profiles for managing genetic diversity, bioinformatic tools are needed^15^. To meet this need, Genassemblage 2.0 software was constructed. Its installer, a detailed user guide and examples of input files can be downloaded free of charge from the author's website: https://pracownicy.uwm.edu.pl/d.kaczmarczyk/main_page.htm.

In this paper, we present an example of the use of Genassemblage 2.0 in conservation of the genetic variation of the lake minnow. The software was used to find the four best combinations of spawning pairs and to find the optimal set of individuals for group spawning.

## Material and methods

23 males and 25 females from one Polish population (Guzy; 54° 08′ 44.25′′ N, 18° 19′ 16.87′′ E) were caught using baited traps. Fin clips were taken, and DNA was extracted from all fin samples using a Genomic Mini AX Tissue SPIN DNA Extraction and Purification Kit (A&A Biotechnology, Poland). The extraction procedure was performed following the manufacturer’s recommendations^16^. The integrity of the DNA samples was visually inspected after their electrophoresis in a 1.5% agarose gel stained with ethidium bromide, and DNA yields were quantified by spectrophotometric analysis^15^.

To prepare genetic profiles and assess genetic variation in the fish sampled from the Guzy population and in groups of their progeny, 13 microsatellites were used as genetic markers. Loci *Ca3*, *Ca4* and *Ca12*^17^, *Z9878*, *Z10362* and *Z13419*^18^ (primer sequences were taken from GeneBank https://www.ncbi.nlm.nih.gov); and *Eupe1*, *Eupe2*, *Eupe4*, *Eupe5*, *Eupe6*, *Eupe7* and *Eupe9* were amplified using primer sequences described by Kaczmarczyk and Gadomski^19^ and deposited in GeneBank (https://www.ncbi.nlm.nih.gov). The primer sequences, repeat motifs, their accession numbers and details of PCR amplification were given by Kaczmarczyk and Wolnicki^16^. The forward primer of each primer pair was 5′-end labeled with fluorescent dyes (6FAM, VIC, NED, PET). The lengths of the amplified DNA fragments were determined using an Applied Biosystems 3130 Genetic Analyzer against GS400LIZ size standards. Allele determination was performed using GeneMapper 3.0 software (Applied Biosystems) according to the manufacturer’s recommendations.

Observed heterozygosity (*Ho*), expected heterozygosity (*He*) and the number of alleles (ar), (note that this is not the same as allelic richness (Ar)) at a given locus and across loci were calculated for both groups of fish using MSA software^20^.

### Genassemblage modules and settings

**Figure 1:**
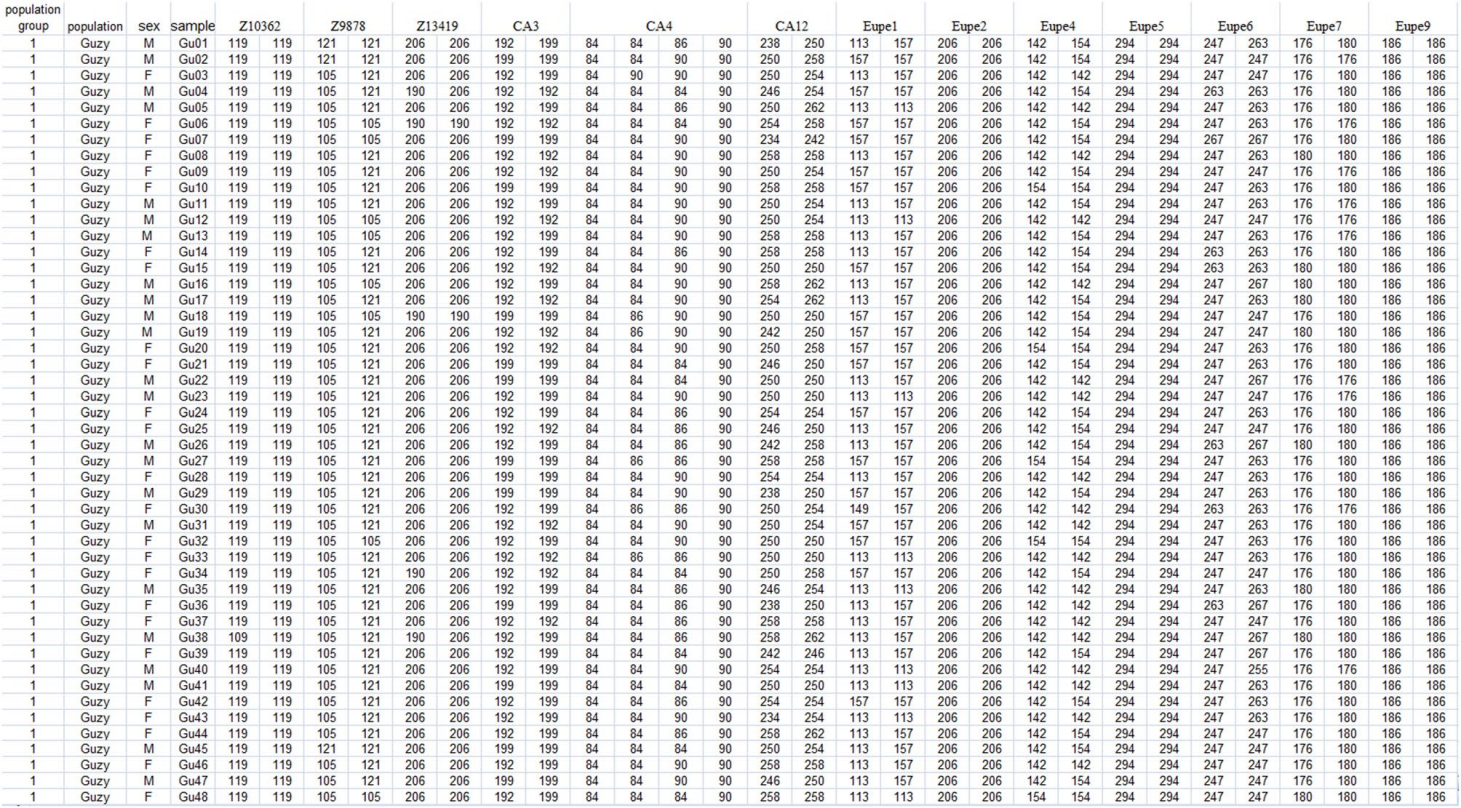
Genetic profiles of lake minnow individuals from the Guzy population (example of input file to Genassemblage 2.0). Column 1, population group; column 2, population name; column 3, sex; column 4, fish ID (samples); columns 5–33, names of microsatellite markers (line 1) and their alleles (lines 2–50).

The Genassemblage 2.0 input file shown in Fig. 1 was used for the calculations described below. The module *Select the best breeding pair and select individuals for group spawning* was used to find the four and then the eight best spawning pairs among all possible combinations.

The best possible breeding pairs for controlled mating were identified based on the heterozygosity, share of “weak heterozygous genotypes” (fish with only one different allele at one locus, for example AAAB)^21^ and allelic diversity expected in progeny, as well as the *v* index, which is a user-defined index for selecting breeding pairs (https://pracownicy.uwm.edu.pl/d.kaczmarczyk/genasemblage2/Genassemblage_2.0_manual.pdf). The coefficients used for calculating the *v* index were a relative index of heterozygosity (*i*_*H*_) 0.45, a share of weak heterozygotes (*i*_*wh*_) 0.1, and a relative index of allelic number (*i*_*ar*_) 0.45; the number of breeding pairs was set at four and then at eight.

To find the optimal set for volitional group-spawning, the module *Find best set of individuals for group spawning* was used. One set consisted of four males and four females, and the other, of eight males and eight females. The number of alleles was selected as the primary indicator. The calculation performed by this module is based on the assumption that each spawner contributes equally to the progeny.

### Ethical statement

These studies were approved by the Local Committee for the Experiments on Animals in Olsztyn, Poland (permission No. 15/2015) issued on 25.03.2015. All procedures were performed in accordance to relevant guidelines.

## Results

In the group of broodstock candidates, 33 alleles were identified across all microsatellite loci. The *H*_*o*_ was 0.386, *H*_*e*_ was 0.375, and the share of weak heterozygotes was 0.208.

With this group of male and female candidates, it was possible to assemble breeding pairs in 575 different combinations. The progeny of each potential pair would have inherited from 17 to 26 (mean of 21) alleles. This is lower than the 33 alleles that were identified in the parental group. Across the investigated markers, heterozygosity ranged from 0.192 to 0.519 (mean 0.378), but only in 211 pairs (36.7%) was the observed heterozygosity higher than that in the parental group as a whole. The average share of weak heterozygotes in groups of progeny was 0.365, ranging from 0.167 to 0.500. The value of this indicator was less than or equal to that in the parental group in only 65 progeny groups (11.3%).

**Figure 2:**
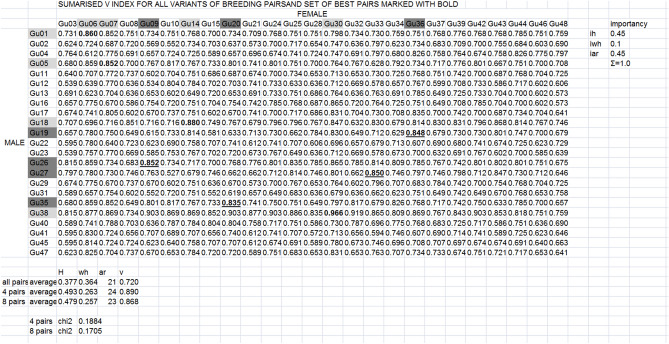
The sets of four and eight best breeding pairs identified by using the *v* index. ID of males are given in column 2, and those of females in line 3. Values of the *v* index are given in lines 4–26 and columns 3–28. The names males and females included in for sets of four and eight pairs are marked in light grey. Their *v* values are marked in bold. The males and females included in set of eight best pairs only are marked in dark grey, their *v* values are marked in bold and underlined. The mean values of *H*, *wh*, *ar*, and *v* index for selected pairs is compared to the means for all pairs in lines 29–31. The importance of each component of the *v* index is given in column 31. The χ^2^ values are given in line 33–34.

Based on the *v* index, a set of four pairs were selected as the optimal pairs for breeding: male Gu01 and female Gu06, male Gu05 and female Gu07, male Gu18 and female Gu14, and male Gu38 and female Gu30 (Fig. 2). The average heterozygosity of this set was 0.493, the number of alleles was 24, the share of weak heterozygotes was 0.263, and the *v* index of this set was 0.890. The *v* index also was used to select a set of the eight best pairs. This set included the four same pairs as listed above and four others that were added to set of four males and four females. They were: male Gu19 and female Gu36, male Gu26 and female Gu09, male Gu27 and female Gu33, and male Gu35 and female Gu20. The average heterozygosity of this set was 0.479, the number of alleles was 23, and the share of weak heterozygotes was 0.257. The *v* index of this set was 0.868 (Fig. 2). All these values of genetic diversity indicators were higher than the averages for all potential breeding pairs (0.377, 21, 0.364, and 0.720, respectively).

**Figure 3:**
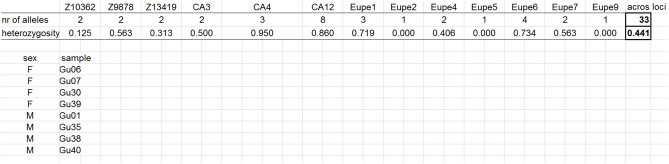
The set of four males and four females for volitional breeding. Line 1—the names of microsatellite markers, lines 2–3—the number of alleles inherited by progeny of this set and their heterozygosity at each locus. Column 30—mean heterozygosity and number of alleles across loci are in bold. Lines 5–13 fish IDs (samples) included in this set (column 2) and their sex (column 1).

**Figure 4:**
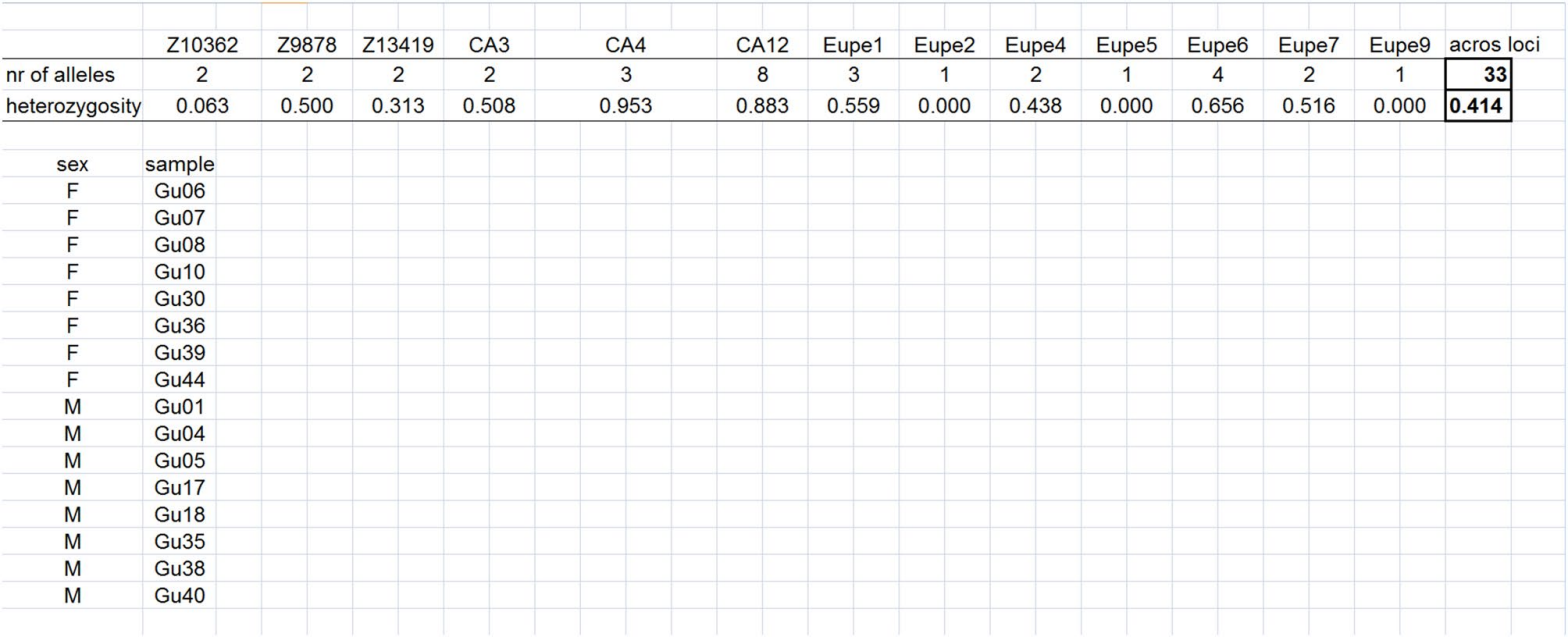
The set of eight males and eight females for volitional breeding. Line 2—the names of microsatellite markers, lines 3–4—the number of alleles inherited by progeny of this set and their heterozygosity at each locus. Column 30—mean heterozygosity and number of alleles across loci are in bold. Lines 6–22 fish IDs (samples) included in this set (column 2) and their sex (column 1).

For group volitional breeding, the 23 males could have been assembled into 8855 different combinations of four individuals, and the 25 females, into 12,650 such combinations. Together, over 120 million different combinations of males and females could have been created. The best set included the males Gu0, Gu35, Gu38 and Gu40, and the females Gu06, Gu07, Gu30, and Gu39. This group would have enabled the transmission of all 33 alleles and in the presented stock, assuming equal contribution, a heterozygosity value of 0.441 in their progeny (Fig. 3). Thus, this set would have transmitted all of the allelic diversity identified in the group of 48 fish sampled from the Guzy population, and the heterozygosity in their progeny would have been higher than that in the parental group. In addition, 2083 sets of individuals were identified that would have transferred 32 or 33 alleles and whose progeny would have had heterozygosity values ranging from 0.400 to 0.440. Those sets were potential alternatives to the set that we selected. When the number of fish in the set was increased to eight males and eight females, the number of potential sets of females increased to 490,314, and that of males, to 1,081,575. Together, this resulted in 5.30311 × 10^11^ combinations for this set. Among them, a large number of combinations enabled inheritance of all 33 alleles and an average heterozygosity of progeny of 0.414. An example of such a set is given in Fig. 4.

## Discussion

Identification of a group of pairs or sets of group-spawning individuals that are optimal for conservation of genetic variation can be time-consuming and difficult. Manually finding the best of them is problematic because of the complexity of the calculations and the huge number of potential pair variants or set compositions. For this reason, bioinformatic tools can be very helpful. Genassemblage 2.0 is a versatile tool for artificial breeding of conserved species such as the lake minnow. It can be used to identify the best breeding set for either individual or group breeding. This is merely an example of how the software works (although the number of markers should be high enough to track genetic differences both within and between lake minnow populations^16,22^). If any molecular marker is polymorphic and inherited according to Mendelian laws, and numbers can be assigned its their alleles, then it can be used. Genassemblage software does not have a limit at the number of molecular markers that can be used. In this study, we used the polymorphism of microsatellite fragments as a marker of genetic differences between individuals. Markers other than microsatellite fragments such as single nucleotide polymorphism can be used, if numerical values are assigned to their alleles.

Using the example of lake minnow individuals from the Guzy population, we have shown that it is possible to identify genetic differences in the broodstock and find the optimal set of spawning pairs. In Poland, the lake minnow requires active protection measures, which can be accomplished without a decrease in the genetic variation of this species by initiating (or suplementing) existing populations with juveniles originating from aquaculture facilities^13,15,23,24^. To prevent decreases in genetic variation and obtain the most genetically diverse set of juveniles, breeding pairs need to be assembled appropriately.

In the group of potential spawners from the Guzy population, we found that the values of genetic variation indicators, such as heterozygosity, allelic diversity and share of weak heterozygotes, indicate that genetic variation in the group of potential parents is low. This finding is similar to reports on other Polish populations of this species^16^.

Our results indicate that random selection of fish for breeding is likely to result in lower genetic variation in the offspring than in the parents. It is known that using a small number of individuals for breeding without information about their genetic similarity and relationships (random selection) can accelerate a decrease in allelic diversity^25–27^. Consequently decrease of population fitness in the next generation or generations is more likely^28^. In the presented example, we found that using a set of breeding pairs chosen by Genassemblage 2.0 will provide a higher level of genetic variation in the progeny than the mean value obtained with the progeny of all potential breeding pairs. Identification of the best set of breeding pairs using the *v* index can increase heterozygosity and reduce the number of weak heterozygotes in progeny. Moreover, each individual included in the set of breeding pairs proposed by Genassemblage 2.0 appears in only one breeding pair, which reduces the risk of inbreeding in future generations.

It should be noted that, in the example presented above, four breeding pairs were chosen for illustrative purposes only. In a real-world application, it would be better to choose more individuals. Using only a few individuals for breeding is associated with a higher risk of a decrease in *Ne*^27^, inbreeding in future generations and increased genetic drift in the population. It is difficult to indicate exactly how many pairs should be used for breeding, but larger numbers help to conserve within-population genetic variation^9^. Consequently, the example that was presented with eight breeding pairs would be more appropriate for conservation of genetic variation than the set of four pairs, even if the four pairs had slightly better genetic variation indicators. Generally speaking, if enough males or females are available and the same individual is not used more than once, it recommended to mate more pairs than four and use Genassemblage 2.0 software to help find the best ones. Genassemblage 2.0 software can be helpful for volitional breeding. If we can assume that the contribution of each spawner to the progeny is equal or very similar, we can find a set of individuals that will transmit all the alleles identified in a group of potential parents to their progeny. In this situation, it is even possible to obtain progeny with higher heterozygosity than in the parents’ generation. Moreover, the software can indicate alternative sets for volitional breeding that provide a level of genetic diversity similar to that in the best set. This can be useful when it is not possible to use the best set. It should be taken into account that, in volitional mass spawning of some fish species, the parental contribution to the progeny can be highly skewed^29–35^. However, this has not been demonstrated with the lake minnow, and our results suggest that using larger sets of males and females in volitional breeding could be more suitable than using separate pairs. Again, it should be noted that, although the average predicted heterozygosity would be slightly lower with eight males and eight females than with four of each, it would be better to use the larger set for the reasons given in the preceding paragraph.

The previous version of the software (Genassemblage 1.0) accurately predicted genetic variation in the progeny of breeding pairs^36^. In comparison, Genassemblage 2.0 has expanded functionality and can be used for more diverse tasks, such as group breeding. Moreover, the calculation of the *v* index, which was implemented in Genassemblage 2.0, enables comparison and evaluation of each breeding pair and selection of the best one. Genassemblage 2.0 has a modular interface, expanded functionality and a more user-friendly design, which makes it even more useful and easy-to-use in conservation of genetic variation. Importantly, the use of Genassemblage 2.0 software is not limited to fish species. It can be used to manage the genetic variation of most species for both conservation and commercial purposes.
